# Deformation and Annealing Behavior of Cr Coating Prepared by Pack-Cementation on the Surface of Austenitic Stainless Steel

**DOI:** 10.3390/ma17143589

**Published:** 2024-07-20

**Authors:** Tongwen Xiao, Jingting Zhang, Fujian Zhang, Huan Su, Jianjun Hu, Ning Guo

**Affiliations:** 1 School of Materials and Energy, Southwest University, Chongqing 400715, China; x17783095077@email.swu.edu.cn (T.X.); scumbag@email.swu.edu.cn (F.Z.); 18996200659@163.com (H.S.); 2 College of Materials Science and Engineering, Chongqing University of Technology, Chongqing 400054, China; zhangjingting@ti-master.cn

**Keywords:** 304 stainless steel, pack-cementation, induction heating, chromizing, cold-rolling, annealing

## Abstract

In this paper, a Cr coating was prepared by induction heating and pack-cementation chromizing on AISI 304 austenitic stainless steel. Then, the cold-rolling deformation and annealing treatment were introduced to refine the coarse matrix grains caused by pack-chromizing and improve the overall performance of 304 austenitic stainless steel. The phase composition, element distribution, and microstructure of the coating were carefully characterized. The microhardness, wear resistance, and corrosion resistance of the coating were tested. The results show that the Cr coating with a thickness of 100 μm is mainly composed of a (Cr,Fe)_23_C_6_, (Cr,Fe)_7_C_3_, and α-Fe-Cr solid solution. After the cold-rolling deformation and subsequent annealing treatment, the grains are significantly refined and the Cr coating is divided into two layers, consisting of carbon-chromium compounds such as Cr_23_C_6_, Cr_7_C_3_, Cr_2_C, and Cr_3_C_2_ in the surface layer and a Fe-Cr solid solution in the subsurface layer. The cold-rolling deformation and annealing treatment significantly improved the microhardness and wear resistance of the coated sample, and the corrosion resistance was also better than that of the uncoated sample.

## 1. Introduction

The 304 austenitic stainless steel is a widely used high chromium–nickel austenitic stainless steel. It has been widely used in various fields because of its good corrosion resistance, heat resistance, and mechanical properties [1,2,3,4,5,6]. However, in high temperatures and complex environments such as nuclear reactors, wear, corrosion, and low hardness problems would accelerate the failure of 304 austenitic stainless steel [7,8]. Improving the surface properties of 304 stainless steel can prolong the service life of the material and adapt to the complex use environment, which has important research value.

Surface coating technology (such as thermal diffusion treatment, vapor deposition process, laser cladding, electroplating, etc.) is one of the most direct and effective methods for improving the surface performance of materials [9,10,11,12,13,14]. Powder pack-cementation technology is widely used because it can achieve good bonding between the coating and the substrate and is simple to operate [15,16,17,18]. Zeng et al. [19] prepared a Si-Cr coating on the surface of AISI 5140 steel by pack-cementation, which significantly improved the microhardness and wear resistance of the substrate surface. It is generally believed that the coating prepared by pack-cementation chromizing can significantly improve the wear resistance and corrosion resistance of materials [20,21,22,23,24,25]. Zhang et al. [26] prepared a Cr-AlN gradient composite coating on the surface of Fe-14Cr-18Ni-4Mo-2Nb-3Al alumina-forming austenitic steel using pack-cementation technology, which significantly improved the room-temperature microhardness and high-temperature wear resistance of the material. Yang et al. [27] performed pack-cementation treatment on hot-rolled Fe_40_Mn_20_Cr_20_Ni_20_ HEA and prepared a Cr coating with a BCC structure and diffusion layer on the surface of the HEAs, which significantly increases the hardness and improves corrosion resistance. However, the problem with the pack-cementation chromizing method is that the heating method during the process is usually box furnace heating, and the heating temperature is high and the holding time is long, which may lead to coarse grains, the overheating of the material core structure, and even the deterioration of performance.

Compared with the box furnace heating method, the induction heating method has the advantages of high efficiency and environmental protection [28]. Meanwhile, for the induction heating method, the current density distribution of the AC of the induction coil on the cross-section of the conductor is uneven. The surface current density is the largest and gradually weakens to the center, resulting in the “skin effect” [29,30,31]. This makes the surface temperature of the induction heating sample higher, and the core structure is less affected by induction heating. Hu et al. [32] found that, after the first step of preboronizing on the surface of AISI 5140 steel using the pack-cementation method, for the second step of pack-chromizing, induction heating can form a thicker, denser, and flatter Cr coating compared to box furnace heating, and the efficiency is higher. Therefore, combining the two technologies of induction heating and pack-cementation can effectively solve the problems caused by a high heating temperature and long holding time during the chromizing process, which improves the efficiency of the chromizing process, and reduces the performance degradation of the core structure of the materials. Considering the characteristics of 304 austenitic stainless steel such as low strength, good plasticity, and low stacking fault energy, it is still possible to strengthen the austenitic structure by introducing deformation and the subsequent annealing treatment, and it is also possible to explore whether the binding force between the coating and the substrate prepared by pack-cementation is excellent. Therefore, in this study, the combination of induction heating and pack-cementation can achieve high efficiency and rapid chromization, and the subsequent cold-rolling and annealing treatment can optimize the coating performance, refine the organizational structure, and effectively improve the surface microhardness, wear resistance, and corrosion resistance of 304 stainless steel.

## 2. Experiments

### 2.1. Experimental Materials

**Figure 1:**
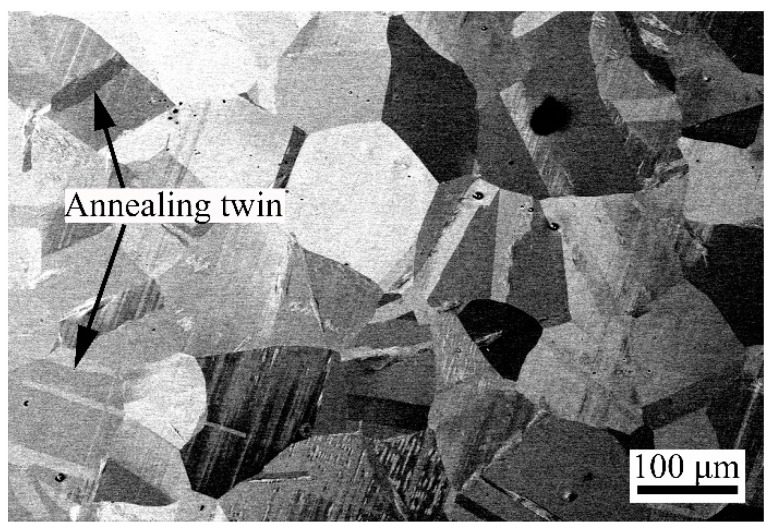
BSEI image of the as-received materials.

In this study, the selected substrate was a commercial 304 austenitic stainless steel (304SS) plate, and the sample size was 25 × 20 × 6 mm^3^. For the pack-cementation coating technology, the size of the samples to be coated can be further enlarged to meet needs. It is necessary to ensure that the sample to be coated can be completely wrapped by the packaging powder and fully heated. The chemical composition (mass fraction) is shown in Table 1. Before the pack-cementation treatment, 304SS was solution-treated at 1050 °C, kept warm for 1 h, and then water-cooled. The original microstructure of the sample after the solution treatment is a single austenite grain. As shown in Figure 1, the measured grain size of austenite is approximately 100 μm, and the austenite structure contains annealing twins.

### 2.2. Coating Sample Preparation and Processing

**Figure 2:**
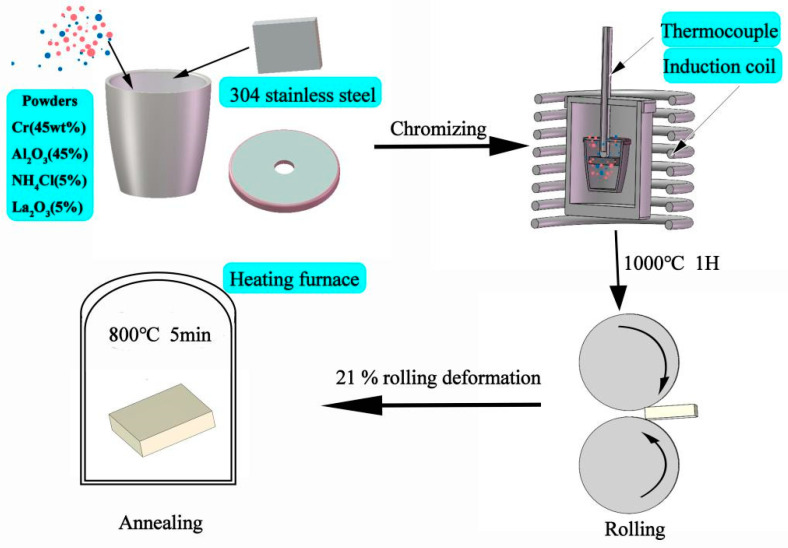
Schematic diagram showing coating preparation and deformation process.

The experimental process used in this study is shown in Figure 2. Prior to the pack-cementation treatment, the samples were ground with 150#, 240#, 400#, 600#, and 800# sandpaper, and the surface of the samples were ultrasonically cleaned by pure alcohol. The packaging powder formula (mass fraction) was: 45% Cr powder as feedstock; 45% Al_2_O_3_ powder as an inert filler; 5% NH_4_Cl as the activator, and 5% La_2_O_3_ as the modifier. After the four kinds of powders were fully mixed evenly according to the proportion, place the packed composite powder and 304SS sample together in a high-temperature-resistant ceramic crucible. The holes on the crucible cover were inserted into the thermocouple, and the mixture of high-temperature refractory mud and water glass was used for the sealing treatment. Then, the mixture was heated to 1000 °C in an induction heating furnace and held for 1 h (the sample is denoted as Coated). Then, the cold-rolling deformation of the 21% rolling deformation was carried out by a rolling mill (the sample was denoted as Coated-CR), and, finally, the annealing treatment at 800 °C (slightly higher than the austenite recovery and martensite inversion temperature [33]) for 5 min was carried out by the box furnace (the sample was denoted as Coated-CR-A), and subjected quickly to water cooling to prevent the obtained fine equiaxed grain’s further growth.

### 2.3. Microstructure Characterization and Property Testing

After pack-chromizing, the sample was cut along the section by wire cutting to facilitate the observation of the interface microstructure. The phase identification of the samples (Coated, Coated-CR, and Coated-CR-A) after pack-chromizing was performed by using an X-ray diffractometer (XRD, DX-2500, Dandong Fangyuan Instrument, Dandong, China). The phases were determined using the International Diffraction Data Center database [34]. The surface and cross-sectional microstructure and corresponding element distribution were characterized by using backscattered electron imaging (BSEI), secondary electron imaging (SEI), energy dispersive spectrometer (EDS, AZtech Max2, Oxford Instruments, London, UK), and electron backscatter diffraction (EBSD, AZtech Max2, Oxford Instruments, London, UK) installed in a field emission gun scanning electron microscope (FEG-SEM, Zeiss Sigma HD, Zeiss, Dresden, Germany). Post-processing of the EBSD data was performed using the Channel 5 software package. A broad-beam argon ion milling and polishing system ion milling (Fischione SEM Mill, Export, PA, USA) were used to polish the sample surface for the EBSD mapping.

The determination of the Vickers hardness across the coating was performed on a Vickers indenter (HVS-1000Z, Shanghai CSOIF Co., Ltd., Shanghai, China) with a load of 2 N and a dwell time of 10 s. The friction resistance was tested by the HSE-2M high-speed reciprocating friction and wear tester (Zhongke Kaihua Science and Technology Development Co., Ltd., Lanzhou, China). The specific parameters were as follows: the hard alloy steel ball (GCr15) with a diameter of 6 mm was used to load 15 N, the duration was 30 min, the frequency was 2 Hz, the wear scar length was 10 mm, and the temperature was room temperature. In a 3.5% NaCl deionized water solution, the potential polarization curve of the sample was tested by a GAMRY electrochemical device (Reference 3000, Warminster, PA, USA), and the corrosion resistance of the coating was evaluated.

## 3. Results

### 3.1. Phase Identification

**Figure 3:**
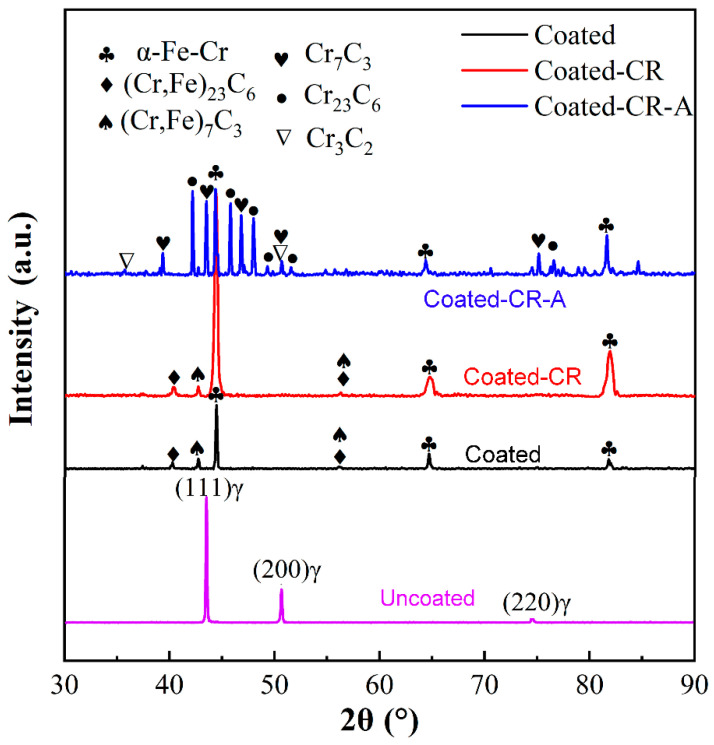
XRD phase profiles of various samples.

Figure 3 shows the XRD patterns of various samples. The initial material (Uncoated) exhibits a single austenitic structure. The surface phases of the Coated and Coated-CR samples are mainly the (Cr,Fe)_23_C_6_, (Cr,Fe)_7_C_3_, and α-Fe-Cr solid solution phases, which is consistent with the results in the literature [28]. The surface phases of the annealed samples are mainly Cr_23_C_6_, Cr_7_C_3_, Cr_2_C, and Cr_3_C_2_. After cold-rolling, a martensite peak appears in the matrix structure of the Coated-CR sample, which is mainly the deformed α’-martensite structure. The martensitic phase has lower free energy than the austenitic phase at room temperature, which is the driving force for the transformation of austenite to martensite during cold deformation [35,36]. Therefore, austenite undergoes deformation-induced martensitic transformation during cold deformation. The transformation of austenite, which is a metastable phase at room temperature, to martensite can be stimulated by thermal driving forces (cryogenic treatment [37]) or mechanical driving forces (deformation [36]). The deformed martensite phase can be reversed into the austenite phase during heat treatment. There is still a certain amount of α’-martensite in the Coated-CR-A sample.

### 3.2. Microstructure Characteristics of the Coating

**Figure 4:**
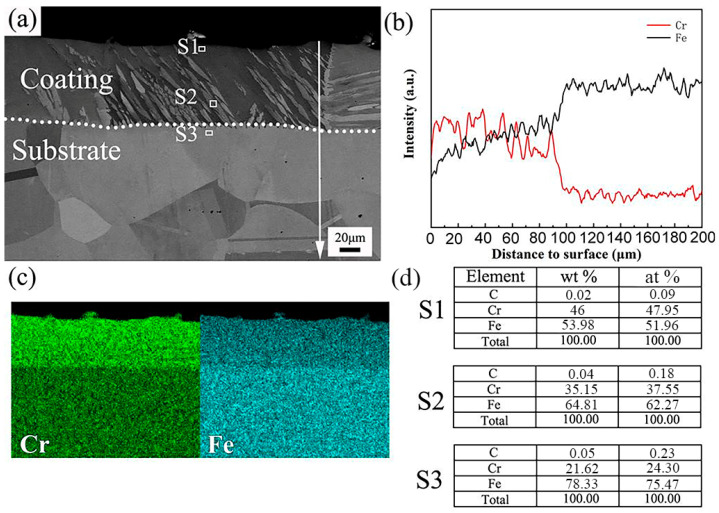
Sectional views showing microstructure (**a**) and EDS results (**b**–**d**) of the coated sample.

Figure 4 displays the cross-sectional microstructure of the Coated sample and the corresponding results of EDS map scanning, line scanning, and point scanning after pack-chromizing. It can be seen that the cross-sectional microstructure of the Coated sample after chromizing is mainly divided into the coating and substrate (see Figure 4a). Through an XRD phase analysis, it can be known that the coating is mainly composed of (Cr,Fe)_23_C_6_, (Cr,Fe)_7_C_3_, and α-Fe-Cr. Among them, (Cr,Fe)_23_C_6_ and (Cr,Fe)_7_C_3_ are mainly columnar crystals precipitated at a certain angle along the grain boundary. The formation of columnar crystals is mainly related to the precipitation of C atoms along the grain boundary from the matrix to the coating. The coating is mainly the α-Fe-Cr solid solution phase. From Figure 4b, it can be seen that the thickness of the coating is about 100 μm, and the Cr element between 90 μm and 100 μm shows a certain gradient decrease, which indicates that there is a certain transition layer between the coating and the matrix. The distribution of Cr and Fe elements in the coating can be seen in Figure 4c. The distribution boundaries of Cr and Fe elements between the coating and the matrix are obvious, and the element drops sharply, indicating that the transition zone between the coating and the matrix is narrow. The point scanning data in Figure 4d are the element contents in the S1, S2, and S3 regions in Figure 4a. The content of Cr (mass percentage) decreases from 44.82% of S1 on the surface of the coating to 20.64% of S3 in the matrix region, indicating that the content of Cr in the coating decreases gradually along the direction of the coating to the matrix, while the content of Fe is the opposite. Compared with the Fe atom, the C atom makes it easier to form carbon-chromium compounds with the Cr atom [28]. In the diffusion process, the activation energy required for lattice diffusion is greater than that for grain boundary diffusion, so the grain boundary is used as a high-speed channel for diffusion. The atomic radius of the C atom is much smaller than that of the Fe atom, and it is easier to diffuse to the coating through the grain boundary in the diffusion process. Due to the low content of the C atom in 304 stainless steel, the carbides in the coating are mainly formed at the grain boundary near the side of the matrix material.

**Figure 5:**
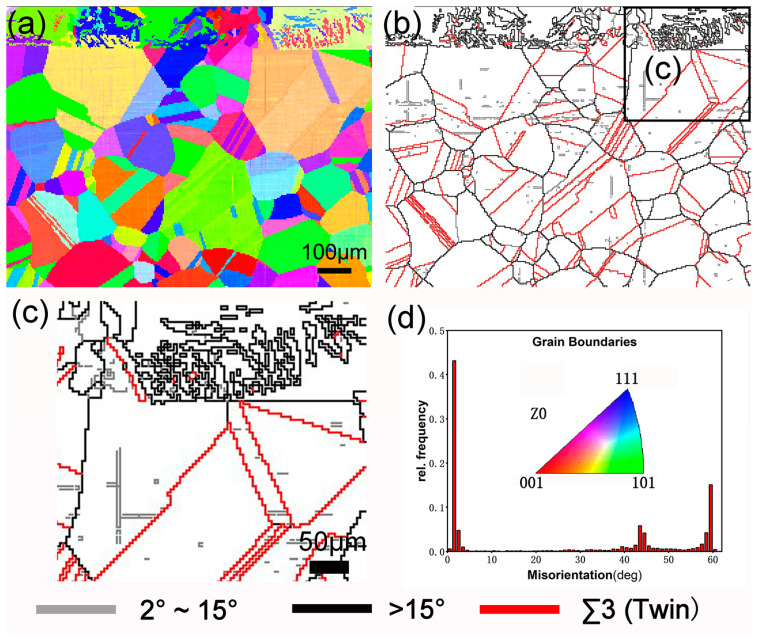
EBSD results show cross-sectional microstructural characteristics of the coated sample: (**a**) IPF map; (**b**,**c**) grain boundary map; and (**d**) misorientation distribution.

To further figure out the change in the microstructure of the coating, the cross-sectional of the induction-heated chromizing sample was characterized by EBSD, as shown in Figure 5. Figure 5a illustrates the inverse pole figure (IPF), and the grain orientation of the Coated sample is relatively uniform without an obvious preferred orientation of a certain crystal plane. Figure 5b is the grain boundary (GB) map. It can be observed that the boundary between the Cr layer and the matrix is obvious. The size of the austenite grain near the coating is bigger than that far from the coating, and the number of twins in the coarse austenite region is less. The austenite grain coarsening is mainly reflected in grain boundary migration, involving thermal activation, diffusion, and interface reactions. Grain coarsening is comprehensively controlled by the driving force and grain boundary movement resistance. During the pack-cementation heating, the growth and coarsening of austenite grains are driven by the reduction in boundary energy. At the same time, there will also be resistance from the diffusion activation energy and carbide particle pinning [38]. The interstitial solid solution and carbide content in austenite are reduced, and the pinning effect is greatly reduced, resulting in the obvious growth of the austenite grain near the Cr coating. Figure 5c is the locally enlarged drawing in Figure 5b. It presents more clearly the bonding area between the coating and the matrix, where the coarse austenite grains are divided by twin boundaries. Figure 5d shows the misorientation distribution and inverse pole figures. It can be seen that the fluctuation of the grain boundary between 2° and 10° in the low-angle boundaries region (2–15°) is obvious, and the low-angle boundaries generated in the austenite matrix organization. The large-angle boundaries region (>15°) is mainly composed of 40–50° grain boundaries and 60° twin boundaries, which are the austenite grain boundary, columnar grain boundary, and twin boundary of Fe-Cr carbide.

After the chromizing treatment, the modified chromizing layer was prepared on the surface of 304 stainless steel, but the matrix grain became coarse due to heat treatment, which reduced its hardness and affected the performance of the matrix material. The microstructure of the matrix is transformed from equiaxed austenite grain with a diameter of 100 μm to coarse austenite grain with a diameter of 100–500 μm. The austenite grains at the interface between the infiltration layer and the matrix are coarser. Coarse grains would lead to a reduction in the hardness and properties of the matrix and a weakening of the bond between the coating and the matrix. Therefore, in this paper, deformation annealing treatment is introduced to solve these defects in the process of chromizing to achieve the effect of grain refinement.

### 3.3. Effect of Cold-Rolling Deformation on Coating

**Figure 6:**
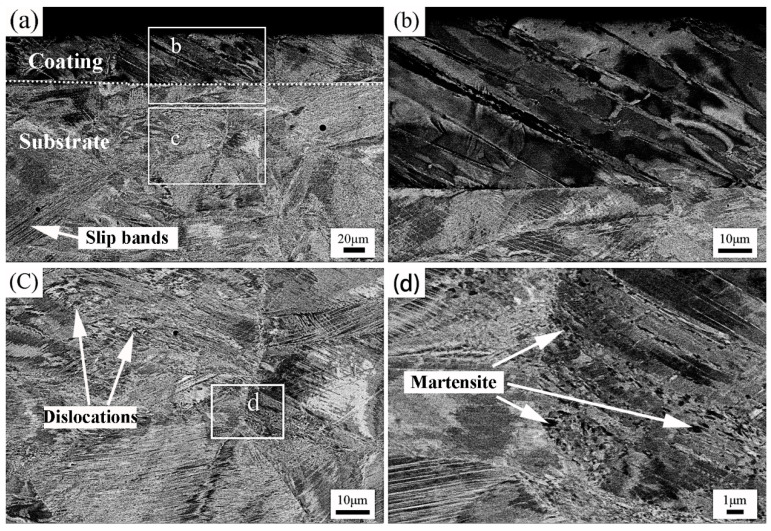
BSEI images showing the cross-sectional microstructure of the Coated-CR sample: (**a**–**d**) different magnification microstructure.

The structure of 304 stainless steel is metastable austenite at room temperature, and the austenite grain is refined during rolling deformation, which will also transform into deformation-induced martensite [39,40,41]. It is reported that deformation-induced martensite has a finer grain size and higher strength than austenite [42], so the rolling deformation will refine the grain and improve the strength of 304 stainless steel. The BSEI images of the 21% rolling deformation sample (Coated-CR) are shown in Figure 6. It can be seen that there are no cracks and defects between the chromized 304 stainless steel and matrix after the cold-rolling treatment (see Figure 6a,b), which indicates that the metallurgical bonding between the coating and matrix is very close. The slip bands appear in the coating at an angle of approximately 45° to the matrix, which indicates that the microstructure of the coating also has a certain deformation in cold-rolling. The slip bands in the austenite grains are interlaced and entangled, and dislocations accumulate here to form deformation-induced martensite. The stress at grain boundaries and slip bands is more concentrated, and the deformation-induced martensite is mainly formed at the grain boundaries and slip bands, which is consistent with the results reported in the literature [43].

After the cold-rolling deformation, the deformation of the coating is compressed and the organizational structure is divided by slip bands. The austenite grains in the matrix structure are divided into irregular fine grains due to the slip bands, and, under the action of the cold-rolling deformation, part of the austenite transforms into α’-martensite. α’-martensite is mainly concentrated in the stress concentration location such as slip bands and grain boundaries. The above effects provide internal stress and a driving force for annealing to refine the grains. It should be noted that the thickness of the Coated sample is reduced due to the cold-rolling deformation, and the thickness of the surface coating is less than 100 μm.

### 3.4. Effect of Annealing Treatment on Coating

**Figure 7:**
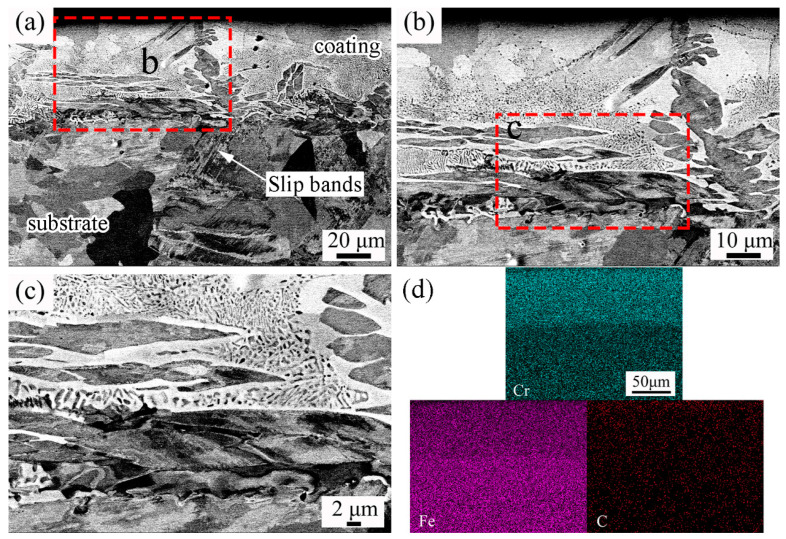
Sectional views showing microstructure (**a**–**c**) and EDS results (**d**) of the Coated-CR sample.

The annealing process is mainly controlled by the annealing temperature and annealing time. The fine equiaxed austenite microstructure can be obtained by the appropriate annealing process parameters to refine the grains and improve the microstructure and properties of the matrix [44]. Figure 7 displays the cross-sectional micromorphology of the Coated-CR-A sample and the corresponding element distribution. The microstructure of the coating is divided into two different morphologies. The coating near the surface is a continuous lamellar structure, and the coating near the matrix is discontinuous fine dendrites and a darker lamellar structure (see Figure 7a). Combined with an XRD phase analysis, it can be known that the continuous layered structure is chromium carbide. There are deformed grains and slip bands after the rolling deformation in the matrix organization, and there are also fine equiaxed crystals produced by recrystallization. It can be seen that, under the experiment conditions of holding at 800 °C for 5 min and rapid cooling, the matrix organization is not completely annealed, which is consistent with the result of XRD that martensite still exists in the matrix. Figure 7b,c are the local magnification of the infiltration layer. The columnar crystal of Fe-Cr carbide similar to the Coated sample appears in the coating (see Figure 7b,c). This is because the C atom diffuses to the coating along the grain boundary during annealing. The grain boundary of the columnar crystal during chromizing and the slip bands generated by cold-rolling provides a channel for the diffusion of the C atom to the coating [45,46]. The Cr atoms combine with diffused C atoms to form carbon-chromium compounds in the coating. During the C atom diffusion process, the subsequent rapid cooling by water terminated the diffusion of the C element and the reaction with the Cr element, so the formed microstructure has the network structure, and fine dendritic precipitation crystals form around the columnar grain boundary (see Figure 7c). In addition, the content of the Cr and C element decreases gradually from the surface to the matrix (see Figure 7d). With the increase in diffusion channels of the C element and the more sufficient combination of the Cr element, compact flake carbon-chromium compounds were formed.

**Figure 8:**
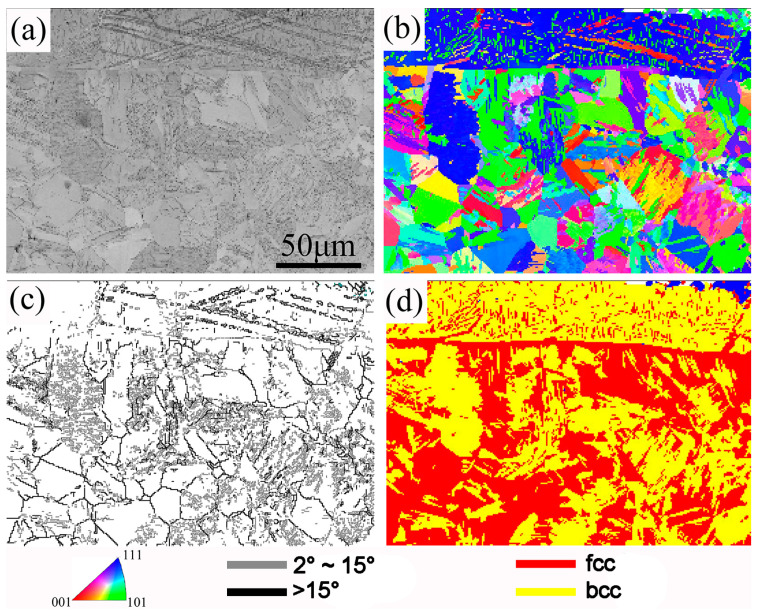
EBSD results show cross-sectional microstructural characteristics of the Coated-CR-A sample: (**a**) band contrast map; (**b**) IPF map; (**c**) grain boundary map; and (**d**) phase map.

To further understand the evolution of the structure and the changes in grains in the Coated-CR-A sample, EBSD testing and analysis of the cross-section of the Coated-CR-A sample were performed and the EBSD results are shown in Figure 8. It can be clearly seen from the BC map that there is still an obvious boundary between the coating and the matrix after annealing (see Figure 8a). It is worth noting that there are still slip bands in the coating, while the slip bands of the matrix are significantly reduced, which indicates that the annealing recrystallization temperature of the coating is higher than that of the matrix. Compared with the grain size of the Coated sample (see Figure 5), the grain size of the Coated-CR-A sample after annealing is significantly refined (see Figure 8b). Especially, there are more fine grains at the stress concentration of the rolling deformation, because the content of α’-martensite at the stress concentration is more. In the annealing process, the deformation-induced martensite transforms into austenite in the inverse phase, and the deformed grains also recrystallize. Due to the short holding time, the equiaxed grains cannot grow and form fine equiaxed grains. Obviously, the grain boundaries in the Coated-CR-A sample are mainly composed of more low-angle boundaries and a small number of large-angle boundaries (see Figure 8c). It can be seen from the grain boundary map that the matrix structure after annealing is not uniform, and some grains are also filled with small gray grain boundaries, because the deformation of this part of the grain in the rolling process is large, and part of the grains have not yet fully recovered during annealing. Some equiaxed grains without low-angle grain boundaries are produced by recrystallization. It can be observed from the phase map that the Coated-CR-A sample mainly consists of fcc and bcc phases (see Figure 8d). The coating near the matrix is mainly the bcc phase, which is the body-centered cubic phase of the α-Fe-Cr solid solution. The content of the fcc phase in the matrix is relatively high and the fcc phase is the austenite phase, indicating that the fine austenite is mainly formed by the recrystallization of deformed austenite and a small amount of α’-martensite reverse transformation.

### 3.5. Mechanism Analysis

**Figure 9:**
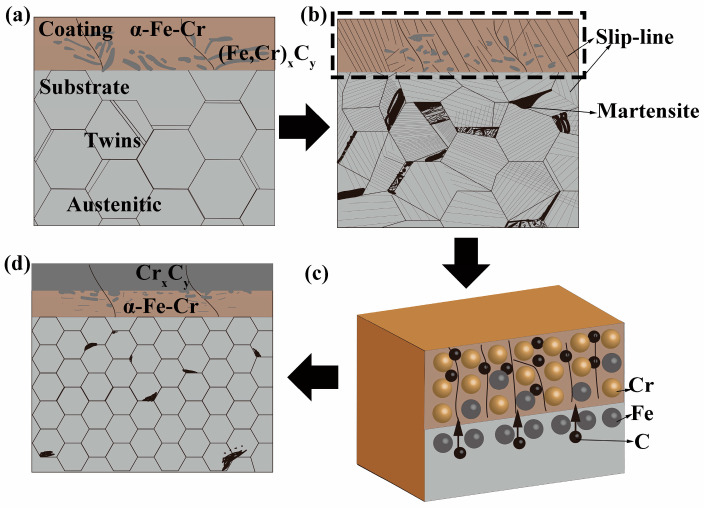
Schematic diagram showing microstructure evolution of pack-cementation Cr-coated austenitic steel during cold-rolling and annealing treatments. (**a**) as coated; (**b**) after cold-rolling; (**c**) diffusion during annealing; (**d**) after annealing.

Through the microstructure analysis of the annealed sample, it can be known that the matrix microstructure of the sample after rolling and annealing is composed of fine equiaxed austenite grains and deformed martensite. The microstructure of the coating is divided into surface-dense lamellar carbon-chromium compounds, and the α-Fe-Cr solid solution near the matrix is body-centered cubic. The combination between the coating and the matrix microstructure is close, without cracks and holes. Figure 9 illustrates the mechanism of the effect of the cold-rolling deformation and subsequent annealing treatment on the coating. Before the cold-rolling deformation, the Coated sample consists of a coating and an austenite matrix (containing a small number of twins), where the coating is composed of (Fe,Cr)_x_C_y_ and Fe-Cr solid solution with a body-centered cubic structure (see Figure 9a). After the Coated sample is deformed by cold-rolling, obvious and more slip bands appear on the surface coating. The matrix undergoes a deformation-induced phase transformation, and part of the austenite transforms into α’-martensite and the grains are refined (see Figure 9b). Figure 9c is a schematic diagram of atomic diffusion during the annealing process in the dotted box area in Figure 9b. It can be seen that the C atom diffuses from the matrix organization to the coating through the slip band and the crystal boundary, and combines with the Cr atom in the coating to form the carbon-chromium compound. As we can see in Figure 9d, after the cold-rolling deformation and subsequent annealing treatment, the surface coating of the Coated sample is divided into two layers, which are the continuous and dense carbon-chromium compound in the surface layer and the Fe-Cr solid solution with a body-centered cubic structure in the subsurface layer. The α’-martensite in the matrix is transformed into austenite during the annealing process but a small amount of α’-martensite is still retained. The austenite grains are equiaxed and the grains do not grow significantly. The carbon-chromium compounds in the coating greatly improve the hardness of the coating, and the matrix structure is also refined and strengthened, so the performance is improved.

## 4. Performance Comparison

### 4.1. Microhardness

**Figure 10:**
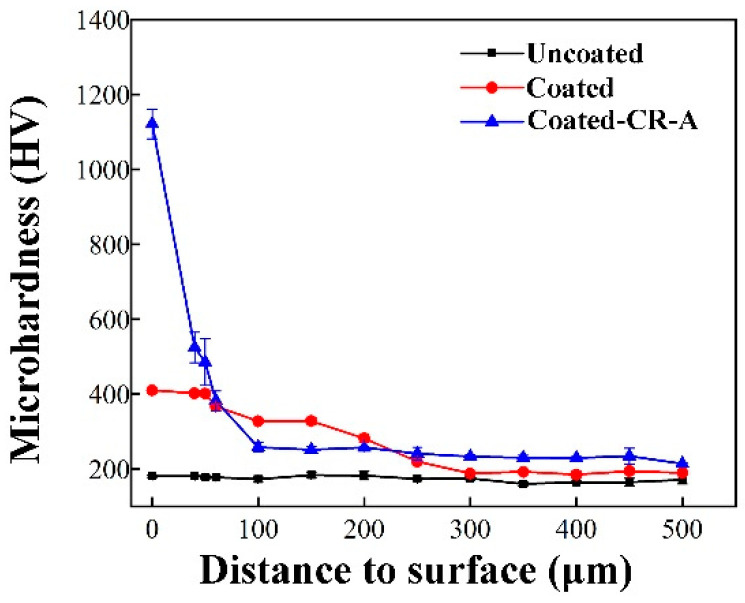
Microhardness distribution of various samples. The error bars represent the standard deviation.

Figure 10 shows the microhardness distribution along the depth direction of 304 stainless steel Uncoated, Coated, and Coated-CR-A samples. It can be clearly seen that the microhardness of the samples (Coated and Coated-CR-A samples) after chromizing presents a gradient distribution along the depth direction. This gradient distribution of hardness is beneficial to the material properties and can make the material have a high external hardness and good internal toughness [47]. The gradient change in microhardness is caused by the concentration gradient of the Cr element in the coating because, in this study, the carbon-chromium compound contributed the most to the improvement in microhardness. The microhardness of the Coated sample is approximately 400 HV, which is due to the low content of the C element in 304 stainless steel, and the lack of the C atom diffusion channel in the coating and the carbide formed cannot be evenly distributed on the surface coating. The coating after cold rolling and annealing is a dense carbon-chromium compound layer, which greatly improves the microhardness, and its hardness is 1120 HV. Meanwhile, the grains are refined in the matrix. Under the action of fine grain strengthening, the microhardness of the Coated-CR-A sample matrix is about 40 HV higher than that of the Uncoated sample.

### 4.2. Wear Resistance

**Figure 11:**
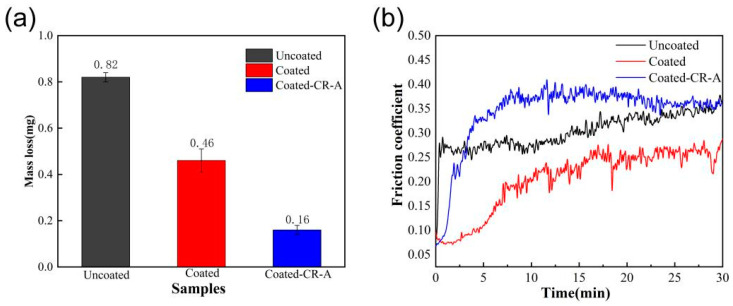
Room temperature friction and wear properties of various samples: (**a**) Mass loss; (**b**) friction coefficients.

Figure 11 displays the wear amount and friction coefficient of Uncoated, Coated, and Coated-CR-A samples. As shown in Figure 11a, the wear amounts of Coated and Coated-CR-A samples are only 0.46 and 0.16 mg, respectively, which is much smaller than the Uncoated sample (0.84 mg). It indicates that the Cr coating in this study significantly improved the wear resistance of the material surface. Figure 11b presents the friction coefficient curves of the above three samples as a function of time. The friction coefficient of the Uncoated sample increases rapidly after contact with the friction pair, and then begins to stabilize. The friction coefficient of the Coated and Coated-CR-A samples increases slowly after contact with the friction pair and gradually stabilizes after about 10 min. This is mainly due to the high hardness of the Cr coating. The average friction coefficient of the Uncoated sample is 0.303, that of the Coated sample is 0.206, and that of the Coated-CR-A sample is 0.344. Generally speaking, when the friction coefficient is reduced, the wear resistance can be enhanced [26,32]. The friction coefficient of the Coated sample is lower than that of the Uncoated sample, and the Coated sample has better wear resistance. However, the friction coefficient is not completely positively related to the wear resistance. After the Coated-CR sample is annealed (without atmosphere protection or vacuum), the surface of the material is oxidized, the surface roughness increases, and the lubrication effect becomes worse, resulting in an increase in friction coefficient. However, this does not mean the resistance of the Coated-CR-A sample is worse than that of the Uncoated sample. For example, the brake pad material in automobiles has a large friction coefficient but still has excellent wear resistance. Comparing the wear amount, Coated-CR-A has a much smaller wear amount, so Coated-CR-A has the most excellent wear resistance.

### 4.3. Surface Corrosion Resistance

**Figure 12:**
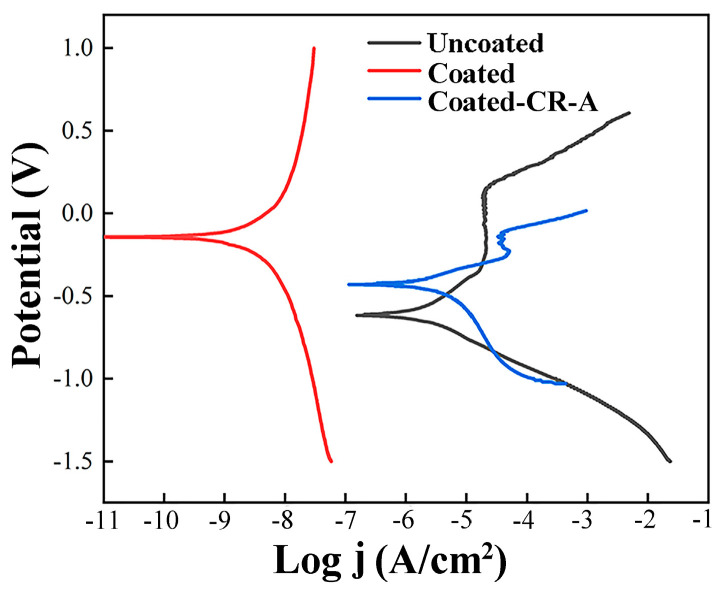
Polarization curves of various samples.

The electrochemical test was carried out in a 3.5% NaCl solution. Figure 12 shows the polarization curves of the Uncoated, Coated, and Coated-CR-A samples. Obviously, the corrosion potential (*Ecorr*) of the Coated sample is the highest, followed by the Coated-CR-A sample, and the Uncoated sample is the smallest. A higher corrosion potential usually means that the corrosion behavior is difficult to start and develop, and the trend of corrosion is inhibited. Table 2 summarizes some important parameters obtained from the electrochemical test, in which the corrosion rate (*CR*) is calculated using the Faraday formula [25], as follows:

(1) $$\mathrm{CR}(\mathrm{mm}/y)=\frac{3.27\times 10^{-3}\times \mathrm{jcorr}\times \mathrm{EW}}{\rho }$$

In this formula, *jcorr* is the corrosion current density (μA/cm^2^), *EW* is the equivalent of the measured material (about 28), and *ρ* is the density of the material (about 7.93 g/cm^3^).

The smaller the value of the corrosion current density (*jcorr*) is, the lower the corrosion rate is. The lower the current density, the lower the rate at which metal materials are damaged [48]. The corrosion potentials of the three samples are −618, −159, and −430 mV, respectively. The corrosion current densities are obtained at the point where the anode Tafel curve and the cathode Tafel curve intersect, which are 3.06, 0.189, and 6.26 μA/cm^2^, respectively. The corrosion rates of the three samples were calculated to be 0.0353, 0.0022, and 0.0723 mm/y, respectively. The corrosion rate of the Coated sample is the lowest. From the above results, it can be concluded that the corrosion resistance of the Coated sample is the best. The Cr element plays a key role in improving corrosion resistance. The dense Cr_2_O_3_ film formed in the passive film of stainless steel plays a major role in corrosion resistance [49]. Compared with the Uncoated sample, the corrosion rate of the Coated-CR-A sample after corrosion is faster than that of the Uncoated sample. This is mainly because the grain of the coating after the cold-rolling and annealing treatment is refined, and the increase in grain boundary leads to an increase in the corrosion rate. Even though the corrosion jcorr and corrosion rate are the highest, the corrosion potential of the Coated-CR-A sample is larger, indicating that the Coated-CR-A sample has a higher threshold at which corrosion begins and it is more difficult for corrosion to occur. This improves the corrosion resistance to a certain extent.

## 5. Conclusions

In this study, a Cr coating was prepared on the surface of commercial 304 austenitic stainless steel by pack-cementation, and the coated samples were subjected to cold-rolling deformation and the subsequent annealing. The microstructure, microhardness, and wear resistance of Uncoated, Coated, Coated-CR, and Coated-CR-A samples were characterized and tested. The main conclusions are drawn as follows.

(1) By pack-cementation, a continuous and dense Cr coating of approximately 100 μm is formed on the surface of commercial 304 austenitic stainless steel. The Cr coating is mainly composed of (Cr,Fe)_23_C_6_, (Cr,Fe)_7_C_3_, and α-Fe-Cr solid solution.
(2) After the cold-rolling and annealing treatment, the grains are significantly refined, the coating and the matrix are still well-bonded, and the coating is divided into two layers. The surface layer is composed of carbon-chromium compounds such as Cr_23_C_6_, Cr_7_C_3_, Cr_2_C, and Cr_3_C_2_, while the subsurface layer is composed of a single uniform Fe-Cr solid solution with a body-centered cubic structure.
(3) The concentration gradient distribution of the Cr element in the coating leads to the gradient distribution of microhardness, which makes 304 austenitic stainless steel a functionally graded material with a high external hardness and good internal toughness. The Cr coating significantly improves the microhardness, wear resistance and corrosion resistance.

## Figures and Tables

**Table 1:** Chemical composition (wt.%) of the austenitic stainless steel.

C	Si	Mn	Cr	Ni	S	Fe
0.082	0.517	1.208	18.216	8.006	0.002	Bal.

**Table 2:** Electrochemical parameters of various samples.

Samples	*Ecorr* (mv)	*jcorr* (μA/cm^2^)	*CR* (mm/y)
Uncoated	−618	3.06	0.0353
Coated	−159	0.189	0.0022
Coated-CR-A	−430	6.26	0.0723

## Data Availability

The raw data supporting the conclusions of this article will be made available by the authors on request.
